# Early Child Development Outcomes of a Randomized Trial Providing 1 Egg Per Day to Children Age 6 to 15 Months in Malawi

**DOI:** 10.1093/jn/nxaa088

**Published:** 2020-04-14

**Authors:** Elizabeth L Prado, Kenneth Maleta, Bess L Caswell, Matthews George, Lisa M Oakes, Michaela C DeBolt, Megan G Bragg, Charles D Arnold, Lora L Iannotti, Chessa K Lutter, Christine P Stewart

**Affiliations:** Department of Nutrition and Institute for Global Nutrition, University of California Davis, Davis, CA, USA; School of Public Health and Family Medicine, University of Malawi College of Medicine, Blantyre, Malawi; Department of Nutrition and Institute for Global Nutrition, University of California Davis, Davis, CA, USA; School of Public Health and Family Medicine, University of Malawi College of Medicine, Blantyre, Malawi; Department of Psychology and Center for Mind and Brain, University of California Davis, Davis, CA, USA; Department of Psychology and Center for Mind and Brain, University of California Davis, Davis, CA, USA; Department of Nutrition and Institute for Global Nutrition, University of California Davis, Davis, CA, USA; Department of Nutrition and Institute for Global Nutrition, University of California Davis, Davis, CA, USA; Brown School, Institute for Public Health, Washington University in St. Louis, St. Louis, MO, USA; RTI International, Washington DC, School of Public Health, University of Maryland, College Park, MD, USA; Department of Nutrition and Institute for Global Nutrition, University of California Davis, Davis, CA, USA

**Keywords:** eggs, child development, complementary feeding, motor, language, memory, eye tracking

## Abstract

**Background:**

Eggs are a rich source of nutrients important for brain development, including choline, riboflavin, vitamins B-6 and B-12, folate, zinc, protein, and DHA.

**Objective:**

Our objective was to evaluate the effect of the consumption of 1 egg per day over a 6-mo period on child development.

**Methods:**

In the Mazira Project randomized controlled trial, 660 children aged 6–9 mo were randomly allocated into an intervention or control group. Eggs were provided to intervention households during twice-weekly home visits for 6 mo. Control households were visited at the same frequency. At enrollment, blinded assessors administered the Malawi Developmental Assessment Tool (MDAT), and 2 eye-tracking tasks using a Tobii-Pro X2–60 eye tracker: a visual paired comparison memory task and an Infant Orienting with Attention task. At endline, 6-mo later, blinded assessors administered the MDAT and eye-tracking tasks plus an additional elicited imitation memory task.

**Results:**

At endline, intervention and control groups did not significantly differ in any developmental score, with the exception that a smaller percentage of children were delayed in fine motor development in the intervention group (10.6%) compared with the control group (16.5%; prevalence ratio: 0.59, 95% CI: 0.38–0.91). Among 10 prespecified effect modifiers for the 8 primary developmental outcomes, we found 7 significant interactions demonstrating a consistent pattern that children who were less vulnerable, for example, those with higher household wealth and maternal education, showed positive effects of the intervention. Given multiple hypothesis testing, some findings may have been due to chance.

**Conclusion:**

The provision of 1 egg per day had no overall effect on child development in this population of children, however, some benefits may be seen among children in less vulnerable circumstances. This trial was registered at clinicaltrials.gov as NCT03385252.

## Introduction

An estimated 149 million children are stunted and 250 million are estimated to be at risk of not fulfilling their developmental potential, partly due to inadequate dietary intake (1), which is especially likely to occur from the age of 6 to 24 mo, as children transition from exclusive breastfeeding to sharing household meals. During this complementary feeding period, infants require nutrient-dense foods to complement breast milk and support healthy growth and development.

Eggs are an important source of nutrients essential for healthy growth and brain development, such as choline, riboflavin, vitamins B-6 and B-12, folate, zinc, protein, and DHA (2). A recent randomized trial in Ecuador (the Lulun Project) provided 1 egg per day for 6 mo to young children during the early complementary feeding period and found that compared with children who did not receive eggs, the intervention group showed significantly higher linear growth (3), and serum choline and DHA concentrations (4). Although this study did not measure developmental outcomes, it is possible that the intervention could have positively affected brain development through increases in these critical nutrients.

The Mazira Project was a randomized controlled trial in rural Malawi that aimed to replicate the design of the Lulun Project in a different context. The primary objective was to evaluate the effect of the daily consumption of 1 egg over a 6-mo period on child growth among 660 children aged 6–9 mo at baseline. A second objective was to determine whether egg consumption positively affects child development. Analysis of the growth outcomes revealed a significant positive effect on head circumference z-score (HCZ), but no effect on length-for-age z-score (LAZ) or other growth outcomes (5). Here, we report the developmental outcomes of the Mazira Project.

Evaluating the efficacy of early-life interventions requires objective and sensitive measures of infant cognition, during a period when infants have a limited behavioral repertoire. Measures of infant looking behavior may be more sensitive than global developmental measures (6). Infants’ eye gazes provide meaningful information about their cognitive processing. For example, an infant's novelty preference (NP), demonstrated by looking longer at a new picture compared with a previously seen picture, shows that the infant remembers the previously seen picture (7). Researchers have used measures of infant looking behavior by manual coding from videos to evaluate the effects of infant feeding on cognition, for example, the effects of fortifying infant formula with DHA (8). Automated eye trackers are an alternative method to manual coding. These devices detect the gaze focal point using an infrared light source and a set of cameras that capture the light reflected from the cornea. Recent advances have made this technology more feasible for use in low- and middle-income country settings (9, 10). However, automated eye tracking has not yet been applied to assess the outcome of a trial in a low- or middle-income country. In this study, we used automated eye tracking to assess infant cognition, in combination with commonly used developmental assessments based on the acquisition of behavioral milestones.

## Methods

### Study participants and design

This study was an individually randomized controlled trial (clinicaltrials.gov: NCT03385252) conducted in a rural area of the Mangochi district, Malawi. Eligible children were those aged 6–9 mo residing in the catchment areas of the Lungwena Health Center and St. Martins Hospital of Malindi. Details of recruitment and inclusion criteria have been published previously (5). All protocols were reviewed and approved by the Institutional Review Boards at the University of California, Davis, and the College of Medicine in Malawi.

At enrollment, which occurred from February to July 2018, participants were randomly assigned to intervention or control groups in a 1:1 allocation ratio in blocks of 10. The random sequence was generated by a researcher independent of the field team. Allocation codes were concealed in sealed, opaque envelopes. After consent and a series of baseline assessments, a study staff member invited caregivers to select and open 1 envelope to reveal the child's allocation code.

The intervention consisted of 1 egg per day for the study child for 6 mo. Participants, household members, and staff members who conducted household visits were not blind to intervention group allocation due to the nature of the intervention. However, staff members who conducted developmental assessments were blind to intervention group allocation. The eggs were delivered 2 times per week by a study staff member during home visits. Each participating household was provided with a storage basket for the eggs, information about hygiene and handwashing during food preparation, recipes and suggestions for how to prepare eggs, and instructions not to share the child's eggs with other family members. Formative research in the study communities revealed that intrahousehold sharing was highly likely, particularly with siblings. Therefore, the family was provided with an additional batch of 7 eggs per week that could be shared with other family members. During the second household visit each week, the staff person administered a 7-d morbidity history and a brief FFQ focused on animal source foods. During the first 2 wk of the trial, a study staff member visited intervention households 4 d each week to provide additional messaging, coaching, and support to promote feeding the child eggs throughout the study. These visits were repeated for 2 d after intervention households had completed 3 mo of intervention to reinforce egg preparation and safe handling messages and encourage continued adherence.

The control group households were also visited twice per week and received messages about hygiene and handwashing during food preparation, but they did not receive eggs or any other foods during the study period. During the course of the trial, control households received participation incentives such as buckets and laundry tubs. At the end of the trial, they received a mixed basket of food items, including eggs. The total value of the incentives and food package matched the value of the eggs provided to the intervention households. During the twice-weekly home visits, the staff person asked the caregiver to recall the child's most recent meal. Similar to the intervention group, morbidity histories and FFQs were also administered on the second visit each week.

### Data collection procedures

At a baseline clinic visit, a staff member conducted a survey asking about each child's characteristics, administered a multipass quantitative 24-h dietary recall, and performed anthropometric measurements. Dates of birth were recorded from the child's clinic card (95.6% of children) or parental recall. Staff members measured hemoglobin (Hb) concentrations (Hemocue 201) and tested for malaria using a rapid diagnostic test (SD Bioline Malaria Ag P.f/Pan). Children with severe anemia (Hb <5 g/dL) or positive malaria test were referred for treatment. A home visit was conducted during the following week to collect household socioeconomic and demographic information, Household Food Insecurity Access (HFIA) status (11) and administer the Home Observation for Measurement of the Environment (HOME) inventory (12).

Six months after enrollment (endline), children were invited back to the study clinic and the 24-h recall, anthropometric measurements, and blood sample collection were repeated. The Family Care Indicators (FCI) interview was also administered at endline (13).

All developmental assessments were conducted at the study clinic site. At baseline and endline, staff members administered the Malawi Developmental Assessment Tool (MDAT) (14) and 2 eye-tracking tasks. Participants enrolled before 4 April, 2018 were tested on the pilot version of the eye-tracking tasks at baseline (39%). After reviewing the pilot data, we revised the eye-tracking tasks and administered the final version of the tasks at the remaining baseline visits and all endline visits. The final version is described below and the pilot version in **Supplemental Methods**. Only data from the final version of the task were used in analyses reported here. At endline, staff members also administered an elicited imitation task.

### Selection of developmental domains and assessments

Eggs contain several nutrients that are important for global brain development, such as protein and iron (2). Therefore, we selected 1 test of global development, which was previously shown to be appropriate and valid in the local context: the MDAT (see below). Choline, also highly concentrated in eggs, plays a specific role in the development and function of the hippocampus, underlying declarative memory (15), and in the development of attentional processes (16). Therefore, we selected 2 tests of declarative memory, 1 behavioral task, and 1 eye-tracking task. We also selected an additional eye-tracking task, the Infant Orienting with Attention (IOWA) task, which reflects maturation of the neural circuitry underlying visual attention (17).

### Behavioral developmental assessment methods

The MDAT was designed to assess child development in Malawi, drawing items from several standard tests, such as the Denver Developmental Screening Tool and Griffiths Mental Development Scales, plus additional items drawn from culturally appropriate behaviors. Three subscales assess fine and gross motor and language development by direct observation of the child, and 1 subscale assesses personal-social development via the caregiver interview. The MDAT was previously validated in Malawi, with >94% of items showing high reliability (κ >0.4 for interobserver immediate, delayed, and intraobserver reliability) (14). Neurodevelopmental impairment was defined as whether the child failed 2 items or more in any 1 domain at the chronological age at which 90% of the normal reference population would be expected to pass. Using this definition, the MDAT demonstrated high sensitivity (97%) and specificity (82%) to detect children with neurodevelopmental impairment in Malawi (14). We applied this definition to determine the risk of a neurodevelopmental disorder and calculated continuous raw scores and z-scores based on published norms.

The elicited imitation task measures children's declarative memory (7). Our adaptation of the task comprised 8 items based on previously published versions of this task (18–21). Each item consisted of a set of toys and a sequence of 2 target actions. For example, for 1 item the toys were a ball and a dump-truck and the target actions were *1*) put the ball in the bed of the truck and *2*) dump it out. For each item, children first played with the set of toys for 30 s while the tester recorded any target actions spontaneously performed. The tester then demonstrated the sequence of target actions twice. Either immediately (4 items) or after a delay of mean ± SD of 9 ± 2 min (4 items), the child was given 2 30 s opportunities to imitate the sequence of target actions demonstrated by the tester. The items were adapted to the local context in an iterative series of 3 pilot tests. For further details, see **Supplemental Methods**. Children were scored on how many target actions they performed spontaneously and from memory (maximum 16 points each) and how many action sequences they performed in order (maximum 8 points). The scores show little variance at age 6–9 mo because children perform very few target actions, therefore we administered this test at endline only.

The child's mood, activity level, and interaction with the assessor during each developmental assessment was rated as positive or not positive. For details, see **Supplemental Methods**.

### Developmental assessment quality control

All assessors were required to pass knowledge and practice-based evaluations before administering the tests and interviews. Interscorer agreement, which is the agreement between 2 data collectors independently scoring the same test session or interview, was high for the MDAT (95%), HOME inventory (89%), and elicited imitation task (90%). For further details, see **Supplemental Methods**. We conducted additional training for items that showed low agreement.

### Eye-tracking methods

Two eye-tracking tasks were administered: a visual paired comparison (VPC) task, based on Rose (22), and the IOWA task, based on Ross-Sheehy et al. (17). Each child was assessed using 1 of 2 eye-tracking systems, each of which comprised a laptop, an external monitor mounted on an adjustable arm, a webcam attached to the top center of the monitor, and a Tobii Pro X2–60 eye tracker with external processing unit. For further details, see **Supplemental Methods**.

Each eye-tracking system was placed in a room in the study clinic site fitted with 4 black curtains. The curtains were open when participants entered the room and closed before starting the eye-tracking task, creating a curtained booth that blocked out visual distractions. When the curtains were closed, only the monitor was visible to the mother and child, who were seated in a chair facing the monitor (**Supplemental Figure 1**). The child was placed in an infant carrier worn by the mother to minimize the child moving around on the mother's lap and moving out of range of the eye tracker. The monitor was positioned so that it was ∼60 cm from the child's eyes. Staff members requested that the mother look away from the monitor or close her eyes, to avoid unintentionally directing the child's gaze. A staff member monitored the mother and child during the session on the laptop screen via the webcam and reminded the mother to turn away if she started watching the screen.

A dynamic image appeared before every trial to draw the child's gaze to the center of the screen. In the NP task, this was a black cross that alternated with images of colorful toys, which were presented with a variety of sounds designed to draw the child's attention. In the IOWA task, the central image was a bright yellow dynamic smiley face that loomed from small (0° 52’ width × 0° 57’ height) to large (4° 35’ width × 5° 9’ height) at a rate of ∼1.5 Hz and was accompanied by classical music. A staff member monitored the infant's gaze and pressed a key to advance to the next trial when the infant's gaze was located on the central image.

**FIGURE 1:**
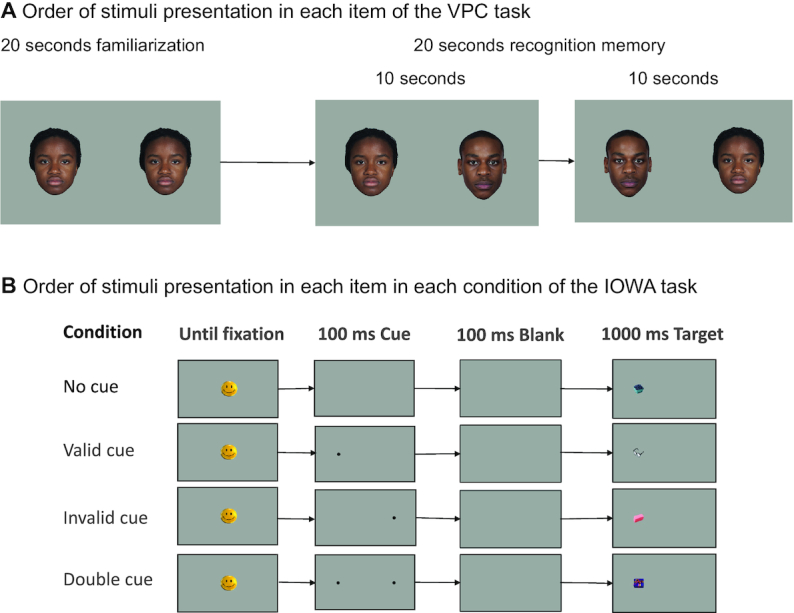
Order of stimuli presentation in the VPC task (panel A) and in each condition of the IOWA task (panel B). All images were presented on a gray background (RGB: 136, 136, 136). In the IOWA task, spatial cues and targets appeared 11° 45’ to the left or right of the central image. The visual attentional cue was a small black circle (0° 56’ diameter). Images were 4° 27’ (w) by 4° 7’ (h) of visual angle. IOWA, Infant Orienting with Attention; ms, milliseconds; RGB, red, green, blue; VPC, visual paired comparison.

The VPC task consisted of 4 trials. In each trial, the stimuli were a pair of African faces from the database reported in Strohminger et al. (23). In each trial a 20 s familiarization period was followed by a 20 s test for visual recognition memory (**Figure 1**A). In each period, 2 faces were presented on the left and right sides of the screen. During the familiarization period, the same face was presented on both sides. During the recognition memory period, the face presented in the familiarization period appeared on 1 side and a novel face appeared on the other side; the stimuli were reversed after the first 10 s. As is typical in eye-tracking tasks to help infants maintain attention and interest in the task in general, unrelated classical music played while the faces were on the screen (24).

In the IOWA task, each trial consisted of the central image, a 100 ms spatial cue, 100 ms blank screen, followed by a target presented for 1000 ms (Figure 1B). Unlike Ross-Sheehy et al. (17), in which cues were presented with a 50-Hz pure tone, in our version of the task no sound was present with the cue. The targets were 96 colorful images of everyday objects, some of which would be familiar to infants and some that would be unfamiliar (e.g. strawberries, stapler, and banana).

Our version of the IOWA task consisted of 3 experimental conditions and 1 control condition (Figure 1B). In the *valid cue* condition, the cue and the target were presented on the same side. In the *invalid cue* condition, the cue and target were presented on opposite sides of the central image (smiley face). In the *double cue* condition, 2 cues were presented, 1 on the left and 1 on the right. The subsequent target then appeared in the spatial location of 1 of the 2 cues. In the *control condition*, no cue was presented; the target appeared without a cue preceding it. Each child saw ≤24 trials in each of these 4 conditions, for a total of 96 trials. Half of the images in each condition were presented on the right side of the screen.

In the testing sequence, VPC trials were intermixed with IOWA trials, in a fixed randomized order. Examples of VPC and IOWA trials are presented in Figure 1. The first VPC trial was followed by 8 IOWA trials (2 in each of the 4 conditions, as defined in the previous paragraph). Then, the second VPC trial was presented followed by 8 IOWA trials, the third VPC trial, 8 IOWA trials, and the fourth VPC trial. Finally, the remaining 72 IOWA trials were presented. The Tobii X2–60 eye tracker recorded the *x* and *y* coordinates of the focal point of the infant's gaze at a rate of 60 Hz (60 times per second). A fixation is a period of time during which the eyes are focused on 1 location. This can be distinguished from eye movement, when the eyes are moving from 1 location to another. We used the Tobii I-VT fixation filter, which is an algorithm built into the Tobii software that classifies the raw eye-tracking data as fixations on various locations.

We examined infants’ looking by creating areas of interest (AOIs), or regions in the display that contained relevant information. A look to an AOI was defined only when that look was classified as a fixation, not when the eyes were moving across the area. In the VPC task, we created AOIs for each face that was presented. Thus, 1 AOI covered the right side of the screen from the right edge of the central image to the right edge of the screen and from top to bottom, and a second AOI covered the mirror image on the left side of the screen. We created conservatively large AOIs to account for poor calibration accuracy, and to mimic the typical VPC data in which human observers simply record whether or not the infant looked to the left or right. We defined an NP score as the child's total time looking at the AOI corresponding to the novel image divided by the total time looking at both AOIs combined during the recognition memory period. To ensure our conclusions were based on trials in which the infants were actually on-task, we excluded trials with <1 s of looking time during either the familiarization or recognition memory periods (11% of trials). We also calculated the child's mean fixation across familiarization periods, excluding fixations <100 ms, which are likely to be artifacts (<1% of trials).

To analyze infant responses to the IOWA task, we created 3 AOIs; 1 around the central image (300 pixels by 300 pixels), 1 covering the left side of the screen, and 1 covering the right side of the screen (similar to the VPC task, described above). A response was scored as correct if the child's first fixation after the onset of the target was to the target side AOI. Trials in which infants’ first fixation after the target onset was to the opposite AOI were scored as incorrect. We determined the response time (RT) on each trial by calculating the time from the onset of the image to the first fixation in the target side AOI.

We excluded any trial in which the child was looking at the central image for <200 ms before the onset of the target image (10% of trials). Because our goal was to evaluate infants’ eye movements from the central image (smiley face) to the target, it was important we only included trials in which infants were actually fixating on the central stimulus. In addition, following Ross-Sheehy et al. (17), we excluded trials with RTs <100 ms and >1000 ms (<1% of trials). RTs 100 ms are too fast to reflect infants’ response to the target, and instead reflect eye movements that began before the target appeared. RTs >1000 ms are too long to reflect infants’ response to the target, and likely represent trials in which the child was off-task. Finally, for the analysis of each condition, we included only those children with data for ≥2 trials. For example, if a child had a single RT in the invalid condition and 2 or more RTs in all other conditions, we excluded that child's scores in the invalid condition only (5% of trials).

We calculated *task error* by averaging percent error in the double and invalid cue conditions, based on Ross-Sheehy et al. (17). We calculated the following scores on correct trials, also based on Ross-Sheehy et al. (17). *Cue facilitation* reflects the degree of facilitation due to the valid cue compared with the no cue condition (i.e. how much faster do infants fixate the target in the valid cue conditions than in the no cue condition). It is calculated as the difference between the mean RT in the no cue and valid conditions, divided by the mean of the no cue condition. *Cue interference* is the degree of interference due to the invalid cue compared with the no cue condition (i.e. how much slower do infants fixate the target in the valid cue conditions than in the no cue condition), and is calculated as the difference between the mean RT in the invalid and no cue conditions, divided by the mean in the no cue condition.

### Statistical analyses

A statistical analysis plan was posted to Open Science Framework (https://osf.io/vfrg7/) before the intervention group code was broken. This plan included prespecified outcomes, covariates, and effect modifiers, as described below. All analyses were conducted using R version 3.5.0 (R Foundation for Statistical Computing). Analysis was by complete case intention to treat. Primary developmental outcomes were the MDAT norm z-scores, elicited imitation total actions recalled, VPC NP score and mean fixation during familiarization, and IOWA RT on correct trials. Secondary developmental outcomes were MDAT raw scores, prevalence of risk of neurodevelopmental disorder, elicited imitation total sequences recalled, and IOWA cue facilitation, cue interference, and task error.

For continuous outcomes, we used linear regression models to estimate the mean difference between groups. For binary outcomes, we estimated prevalence ratios using regression models with a binomial distribution with a log link and prevalence differences using linear probability models. For MDAT and elicited imitation scores, we adjusted for baseline MDAT scores. For eye-tracking outcomes we adjusted for the child's age at assessment, but not baseline eye-tracking scores due to missing baseline data for children tested on version 1. For analyses with repeated trials within participants (VPC NP, IOWA RT), we used robust SEs with participant as the independent unit. For the analysis on NP score, we included familiarization time on each trial as a covariate in all models. For the analysis on IOWA RT, we included a fixed effect of condition.

For each outcome measure, we conducted a secondary adjusted analysis considering a prespecified list of additional covariates, including child sex, age, birth order, maternal age, maternal height, maternal education, maternal literacy, maternal marital status, maternal tribe, maternal occupation, religion, number of children under the age of 5 y in the household, HFIA score, housing and asset index, animal ownership, distance from home to water source, data collector, month of outcome assessment, village location, baseline child LAZ and weight-for-length z-score (WLZ), HOME score, FCI score, time of day of developmental assessment, and child's mood, activity level, and interaction with the assessor during testing. For MDAT language score, we also considered the child's primary language and whether the child was exposed to >1 language. For elicited imitation scores, we also considered the number of spontaneous target actions performed. For the eye-tracking scores, we also considered which of the 2 systems was used for data collection. We prescreened covariates in bivariate models to assess whether they were associated with the outcome prior to including them in the adjusted models. Covariates with a *P* value < 0.1 were included in the analysis. Any variables collected after baseline were only included in the models if they were not different between intervention and control groups.

For the primary eye-tracking outcomes, we conducted an additional analysis controlling for the corresponding baseline scores among the subset of children tested on the final version at both baseline and endline. For further details, see **Supplemental Methods**.

For the primary developmental outcomes, we examined the following 10 potential effect modifiers: child sex, birth order, baseline maternal age and education, baseline HFIA score, baseline housing and asset index, baseline LAZ, corresponding baseline developmental or eye-tracking score below median, HOME score below median, FCI score below median. If any interaction between the potential effect modifier and intervention group was significant at the *P* < 0.1 level, we further explored the pattern of the effect at various levels of the effect modifier.

## Results

**FIGURE 2:**
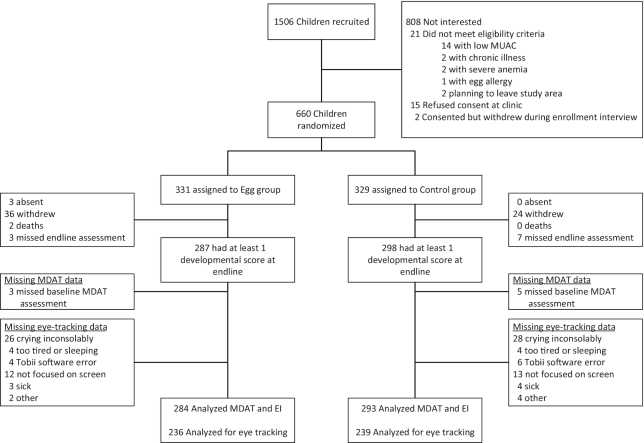
Trial profile. EI, elicited imitation; MDAT, Malawi Developmental Assessment Tool; MUAC, mid-upper arm circumference.

**Figure 2** shows the trial profile. Out of 660 children enrolled, 585 were analyzed for behavioral developmental assessment at endline and 475 were analyzed for eye tracking. A sample size of 585 provided 80% power to detect and effect size of 0.23, and 475 an effect size of 0.26, between 2 groups in continuous outcomes at *P* < 0.05. The losses to follow-up were similar in the intervention and control groups (14% and 11%, respectively, for behavioral developmental assessment and 29% and 27%, respectively, for eye tracking).

Characteristics of the children who were enrolled but did not participate in developmental and eye-tracking assessment are shown in **Supplemental Tables 1** and **2**, respectively. A large proportion of the mothers of children who did not participate in developmental assessment (73%) resided in the Lungwena Health Center catchment area, whereas the loss to follow-up was lower among residents of the Malindi Health Center catchment area. Maternal education was significantly lower among children who did not participate in behavioral developmental assessment. Maternal education and literacy were significantly lower among children who did not participate in the eye-tracking assessment.

**Table 1** presents the characteristics of the developmental sample in each intervention group. Mothers of children in the intervention group were more likely to have completed primary school (25.1% compared with 16.8%). All other characteristics were generally similar.

**TABLE 1:** Characteristics of the children included in the analysis who did (Egg) or did not (Control) receive 1 egg per day for 6 mo^1^

	Control		Egg	
Characteristics	*N*		*N*	
Maternal				
Maternal age, y	298	26.0 ± 6.8	287	26.1 ± 6.6
Maternal BMI, kg/m^2^	298	21.8 ± 2.8	287	21.9 ± 3.4
Maternal education ≥primary, %	298	16.8	287	25.1
Mother can read, %	296	43.2	286	51.0
Maternal marital status, %				
Monogamous	298	59.1	287	54.4
Polygamous		20.8		18.8
Unmarried		20.1		26.8
Child				
Child age, months	298	7.3 ± 1.2	287	7.4 ± 1.2
Female, %	298	47.0	287	48.4
Firstborn, %	298	25.5	286	30.1
Malaria, %	268	13.1	261	12.3
Anemia, %	262	61.5	257	60.3
Breastfeeding, %	298	100.0	286	99.7
Household				
Health center, %	298		287	
Lungwena		50.7		50.5
Malindi		49.3		49.5
HOME inventory score	296	24.0 ± 3.5	286	24.4 ± 3.5
Number of children under 5 y	294	1.7 ± 0.8	283	1.7 ± 0.8
Moderate or severe food insecurity,^2^ %	295	79.9	286	74.6
Own latrine, %	296	97.3	287	95.8
Distance to water source <10 min, %	296	54.4	286	56.8

1 Values are *N*, %, or mean ± SD. HOME: Home Observation for Measurement of the Environment (12).

2 Food insecurity assessed using the Household Food Insecurity Access Scale (11).

The means and prevalence of the primary and secondary developmental outcomes at baseline and endline in the intervention and control groups are shown in **Tables 2** and **3**. At endline, the intervention and control groups did not differ significantly in any continuous MDAT, elicited imitation, or eye-tracking score. For the MDAT, children scored, on average, 0.5 to 1.3 SDs higher than the norm sample, except for language, for which children's scores were similar to the norm sample. MDAT raw scores are shown in **Supplemental Table 3**. For the elicited imitation task, on average, children performed around 7 of the 16 target actions and 1.5 of the 8 sequences.

**TABLE 2:** Primary and secondary developmental outcomes and mean differences between children aged 6–9 mo who did (Egg) or did not (Control) receive 1 egg per day for 6 mo^1^

	Baseline				Endline				Minimally adjusted^2^ comparison (95% CI)	Fully adjusted^3^ comparison (95% CI)
	Control		Egg		Control		Egg			
	*N*		*N*		*N*		*N*			
Primary Developmental Outcomes										
Fine motor norm z-score	323	1.15 ± 1.34	329	1.01 ± 1.17	292	0.52 ± 1.03	283	0.55 ± 1.12	0.07 (−0.11, 0.25)	0.03 (−0.12, 0.19)
Gross motor norm z-score	323	0.79 ± 0.74	329	0.64 ± 0.81	291	0.86 ± 1.30	284	0.70 ± 1.19	−0.08 (−0.28, 0.12)	−0.15 (−0.33, 0.04)
Language norm z-score	323	0.00 ± 0.87	329	−0.04 ± 0.88	293	0.23 ± 0.83	284	0.23 ± 0.82	0.02 (−0.11, 0.15)	−0.05 (−0.18, 0.08)
Personal-social norm z-score	323	1.37 ± 1.03	329	1.36 ± 0.94	293	1.04 ± 1.07	284	1.05 ± 1.08	0.02 (−0.16, 0.19)	0.05 (−0.09, 0.20)
Elicited imitation total actions recalled score	—	—	—	—	293	6.66 ± 3.56	283	6.83 ± 3.56	0.29 (−0.28, 0.87)	0.24 (−0.21, 0.69)
VPC novelty preference score	—	—	—	—	234 (746)	0.59 ± 0.16	225 (689)	0.60 ± 0.16	0.00 (−0.01, 0.02)	0.00 (−0.01, 0.02)
VPC mean familiarization fixation, ms	—	—	—	—	239	380.67 ± 182.14	235	369.64 ± 179.09	−10.96 (−43.56, 21.65)	−8.41 (−40.86, 24.04)
IOWA response time, ms	—	—	—	—	218 (5670)	397.89 ± 176.85	210 (5431)	402.43 ± 177.04	4.82 (−9.72, 19.36)	5.40 (−8.70, 19.50)
Secondary Developmental Outcomes										
Elicited imitation total sequences recalled score	—	—	—	—	293	1.52 ± 1.49	283	1.62 ± 1.55	0.16 (−0.08, 0.41)	0.14 (−0.07, 0.35)
IOWA cue facilitation score	—	—	—	—	189	0.01 ± 0.23	175	0.02 ± 0.25	0.00 (−0.05, 0.05)	0.00 (−0.05, 0.05)
IOWA cue interference score	—	—	—	—	186	0.13 ± 0.27	163	0.14 ± 0.29	0.00 (−0.06, 0.06)	−0.01 (−0.07, 0.05)

1 Values are number of participants (number of trials) included in the analysis, mean ± SD, or mean difference (95% CI).

2 Adjusted for baseline MDAT scores. Eye-tracking measures also adjusted for child age at assessment. VPC novelty preference also controls for familiarization time on each trial in all models. IOWA response time also controls for trial condition in all models.

3 Adjusted for variables in the minimally adjusted model and potentially adjusted for child age at measurement, sex, birth order, maternal age, height, education, literacy, marital status, tribe, occupation, religion, number of children under 5 y in the household, food security, housing and asset index, animal ownership, distance to water source, closest health center, length-for-age z-score, weight-for-length z-score, HOME inventory score, staff member who performed measurements, month of measurement, time of day of measurement, child's demeanor during measurement, and endline family care indicator score. Additionally, the language outcomes are potentially adjusted for the child's primary language and exposure to multiple languages, the elicited imitation outcomes are potentially adjusted for spontaneous actions performed before demonstration, and the eye-tracking measures are potentially adjusted for the eye-tracking system used.

HOME, Home Observation for Measurement of the Environment; IOWA, Infant Orienting with Attention; MDAT, Malawi Developmental Assessment Tool; VPC, visual paired comparison.

**TABLE 3:** Secondary developmental outcomes and prevalence ratios between children aged 6–9 mo who did (Egg) or did not (Control) receive 1 egg per day for 6 mo^1^

	Baseline				Endline				Minimally adjusted^2^ ratio (95% CI)	Fully adjusted^3^ ratio (95% CI)
	Control		Egg		Control		Egg			
	*N*		*N*		*N*		*N*			
Secondary Developmental Outcomes										
Neurodevelopmental delay, %	323	5.0	329	4.3	293	27.1	284	22.3	0.78 (0.58, 1.05)	0.80 (0.61, 1.05)
Fine motor delay, %	323	2.5	329	2.7	293	16.5	284	10.6	0.59 (0.38, 0.91)	0.66 (0.43, 1.00)
Gross motor delay, %	323	0.0	329	0.3	293	0.0	284	0.7	—	—
Language delay, %	323	0.6	329	0.6	293	2.0	284	1.1	0.60 (0.15, 2.41)	0.53 (0.14, 2.07)
Personal-social delay, %	323	1.9	329	1.8	293	12.6	284	12.0	0.91 (0.58, 1.42)	0.77 (0.52, 1.16)
IOWA any task error, %	—	—	—	—	215	61.4	205	67.3	1.09 (0.95, 1.26)	1.14 (0.99, 1.31)

1 Values are number of participants included in analysis, prevalence (percentage of children), or prevalence ratio (95% CI).

2 Adjusted for baseline MDAT scores.

3 Adjusted for variables in the minimally adjusted model and potentially adjusted for child age at measurement, sex, birth order, maternal age, height, education, literacy, marital status, tribe, occupation, religion, number of children under 5 y in the household, food security, housing and asset index, animal ownership, distance to water source, closest health center, length-for-age z-score, weight-for-length z-score, HOME inventory score, staff member who performed measurements, month of measurement, time of day of measurement, child's demeanor during measurement, and endline Family Care Indicator score. Additionally, the language outcomes are potentially adjusted for the child's primary language and exposure to multiple languages, the elicited imitation outcomes are potentially adjusted for spontaneous actions performed before demonstration. HOME, Home Observation for Measurement of the Environment; IOWA, Infant Orienting with Attention; MDAT, Malawi Developmental Assessment Tool.

For the VPC task, children demonstrated a preference for the novel face, with an average of 60% of time spent looking at the novel face compared with the familiar face. Mean fixation during familiarization trials on the VPC task was ∼375 ms. Mean RT on the IOWA task was ∼400 ms. RT on the valid trials was, on average, 1–2% faster than the no cue trials (cue facilitation). RT on the invalid trials was, on average, 13–14% slower than the no cue trials (cue interference). Task error was not normally distributed, therefore we created a binary variable indicating that ∼35% of children committed any error in the double or invalid cue conditions. This prevalence did not differ between intervention and control groups. Results were similar in the analysis controlling for the corresponding baseline scores among the subset of children tested on the final version of the eye-tracking task at both baseline and endline. For further details, see **Supplemental Results**.

A very small percentage of children were classified as developmentally delayed at baseline (overall: 4.6%, fine motor: 2.6%, gross motor: 0.2%, language: 0.6%, personal-social: 1.8%). At endline, the prevalence of developmental delay in gross motor (0.4%) and language (1.6%) remained very low, whereas the prevalence of delay in fine motor increased to 13.6%, delay in personal-social development increased to 12.3%, and overall delay increased to 24.7%. At endline, a significantly smaller percentage of children were classified as delayed in fine motor development in the intervention group (10.6%) compared with the control group (16.5%). No significant differences were found between groups in the prevalence of developmental delay in the other 3 MDAT subscales or overall delay.

Out of the 10 effect modifiers examined for the 8 primary developmental outcomes, 7 (9%) interactions between the effect modifier and group were found to be significant at *P* < 0.05 (**Supplemental Table 4**). This is slightly higher than the percentage that would be significant due to chance (5%), and a consistent pattern emerged that subgroups of children who were less vulnerable at baseline were more likely to show positive effects of the intervention. Positive effects of the intervention were found on fine motor z-scores among children of older mothers (>20 y) (β = 0.21, 95% CI: 0.00, 0.41), children with LAZ > −1 at baseline (β = 0.31, 95% CI: 0.08, 0.54), and children who were not firstborn (β = 0.24, 95% CI: 0.03, 0.44). Positive effects of the intervention were found on language z-scores among children from families with mild to no food insecurity (β = 0.34, 95% CI: 0.00, 0.67) and on personal-social z-scores among children from families in the highest wealth quintile (β = 0.38, 95% CI: 0.00, 0.76). Positive effects of the intervention were found on elicited imitation recall scores among children of mothers with higher levels of formal education (primary or greater) (β = 1.66, 95% CI: 0.49, 2.84). Positive effects of the intervention were found on NP scores among girls (β = 0.02, 95% CI: 0.00, 0.04). The standardized mean difference in scores between groups (presented in units of SD) is shown in **Supplemental Figure 2** stratified for each significant effect modifier.

## Discussion

In this randomized trial in Malawi, an intervention providing 1 egg per day to children for 6 mo during the early complementary feeding period did not affect motor, language, or personal-social development, or memory development measured by an elicited imitation task. In addition, indices of memory and attention derived from infant looking behavior using automated eye tracking did not show effects of the egg intervention. A smaller percentage of children were delayed in fine motor development in the intervention group compared with the control group. Among effect modifiers, we found a consistent pattern that subgroups of children who were less vulnerable at baseline tended to show positive effects of the intervention, for example, those with higher initial LAZ, mild to no food insecurity, higher household wealth, and higher maternal education. A similar pattern was found for the main outcome of the trial, that is a positive effect of the intervention on LAZ among children of mothers with higher maternal formal education (5). Thus, although the primary analyses did not reveal robust effects of the intervention, it is possible that the intervention is effective for this population under some conditions.

One potential explanation for the lack of overall effects of the intervention on child development was that adherence to egg consumption may have been low, especially among more vulnerable children. However, multipass 24-h dietary recalls administered at the baseline, 3-mo, and 6-mo visits suggested that adherence was relatively high (5). According to these recalls, at baseline, only 4% of children consumed eggs on the previous day. In the intervention group, this increased to 85% at the 3-mo visit and 71% at the 6-mo visit, whereas in the control group, the proportion remained low at 6–7% throughout the study (5). Thus, we did not find evidence that low adherence explained the lack of overall effects. Additionally, egg consumption was not associated with factors such as maternal education, food insecurity, and household wealth, suggesting that the positive effects of the intervention found among less vulnerable children cannot be explained by higher adherence among this group.

Another potential explanation for the lack of an overall intervention effect is that other exposures apart from inadequate dietary intake, such as chronic infection and inflammation, may be the major contributors to children's faltering growth and development in this setting. Other studies in the same area have also found no effects of dietary interventions with nutrient-dense foods on children's growth and development (25–29). Improving dietary intake might confer limited benefit if a high burden of infection and inflammation inhibit nutrient uptake. It is also possible that children in more favorable circumstances (higher maternal education, household wealth, etc.) may have a lower burden of infection and inflammation and, therefore, may have greater potential to respond to a dietary intervention. Future analyses of the data from this study will examine morbidity symptoms, markers of inflammation, and dietary practices in order to explore these possibilities.

Finally, we observed that fish consumption was relatively common among the children enrolled in the study. Nearly two-thirds of children in our study were reported to consume fish on the previous day at the 3-mo and 6-mo visits. Dietary interventions with animal source foods may confer limited added benefit on child growth and development in contexts where other high-quality animal source foods are regularly fed to children.

Eggs are an important source of choline, with a higher concentration per gram than any other food except liver or kidneys (30). Choline is necessary for multiple aspects of brain development and function, for example through its role in the structural integrity and signaling functions of cell membranes. Choline plays an especially important role in the development and function of the hippocampus, which underlies declarative memory (30). Choline is a structural component of acetylcholine, a neurotransmitter necessary for the normal formation of declarative memories. In animal models, prenatal choline deficiency has long-term effects on hippocampal function and offspring memory performance (31). We selected the VPC and elicited imitation tasks for this study due to evidence that these tasks assess the developing declarative memory system and its neural substrates, including hippocampal function (7). Although we did not find effects of the intervention on these tasks, future analyses from this trial will examine associations of plasma choline concentrations with these scores.

Our study was the first to use automated eye tracking to evaluate the effect of a randomized trial on infant cognition in a low- or middle-income country. At the beginning of the study, the project experienced negative perceptions in the community of the eye tracking procedure. We therefore expanded the initial participant information that we provided, including gathering participants in the eye-tracking room, explaining in detail the purpose of each piece of equipment, and showing the caregivers the video their children would later view during the eye tracking task. After these changes, perceptions and acceptance of the procedure greatly improved. We demonstrated that this method was feasible for measuring the outcomes of a large field trial in rural Malawi. We successfully obtained usable data from 60% of targeted children aged 6–9 mo and 72% of targeted children aged 12–15 mo. These success rates were obtained in the context of a full day of data collection and project activities for the participants, with as many as 25 participants assessed on any given day. The higher success rate at endline probably reflects both the improvement of the field team with practice and the relative ease of obtaining usable data from slightly older children. Both tasks showed expected patterns of results compared with studies conducted in high-income countries: for the VPC task, children showed the expected preference for the novel face (22), and for the IOWA task, children showed the expected pattern of faster responses on the valid cue trials compared with no cue (cue facilitation) and slower responses on the invalid trials compared with no cue (cue interference) (17). With further effort to increase success rates, these may be promising methods for future research.

Strengths of the study were the randomized design, large sample size, evidence for high adherence based on 24-h dietary recalls, rigorous quality control for data collection, and use of innovative and hypothesis-driven developmental assessments that were appropriate for the local context. The developmental assessment staff were blinded to intervention group, the statistical analysis plan was prespecified and publicly posted, and all analyses were developed on blinded data sets. Therefore, the risk of bias was low. Although attrition for the MDAT and elicited imitation assessments was low, attrition for the eye-tracking assessments was slightly higher due to missing data. In addition, attrition was unbalanced such that those lost to follow-up had lower maternal education. Due to this imbalance, we would be cautious to conclude that the intervention had positive effects, however, our results do not suggest any robust pattern of positive effects. Other limitations were that egg consumption was reported, not directly observed, and households who received eggs may have chosen to share or sell them. However, the provision of 7 eggs per week for the household in addition to 7 eggs per week for the study child was designed to mitigate such sharing.

In summary, we found that provision of 1 egg per day for 6 mo during the early complementary feeding period did not affect child development in the study area in rural Malawi. Although less vulnerable groups of children may have greater potential to respond to an egg intervention, children whose diet already contains animal source foods may not have substantial potential to benefit. This study highlights the importance of considering context when deciding whether to invest in policies and programs to promote child egg consumption. Further research is needed evaluating the effects of eggs on children's growth and development in settings where children are not commonly fed animal source foods and where stunting prevalence is high. Automated eye tracking is a promising new method to evaluate child development in low- and middle-income countries.

## Supplementary Material

nxaa088_Supplemental_File

## Notes

This publication is based on research funded by a grant to the University of California, Davis, from the Bill & Melinda Gates Foundation (OPP1169033). This work was also supported by the USDA National Institute of Food and Agriculture, Hatch project #CA-D-NTR-2493-H.

Author disclosures: The authors report no conflicts of interest.

Deidentified data described in the manuscript, code book, and analytic code will be made available upon request pending approval by the first, second, and last author (ELP, KM, and CPS) and signed material transfer agreement between UC Davis or the University of Malawi and the requesting institution.

Supplemental Methods, Supplemental Tables 1–4, and Supplemental Figures 1 and 2 are available from the “Supplementary data” link in the online posting of the article and from the same link in the online table of contents at https://academic.oup.com/jn/.

Abbreviations used: AOI, area of interest; FCI, Family Care Indicators; Hb, hemoglobin; HCZ, head circumference z-score; HFIA, Household Food Insecurity Access; HOME, Home Observation for the Measurement of the Environment; IOWA, Infant Orienting with Attention; LAZ, length-for-age z-score; MDAT, Malawi Developmental Assessment Tool; NP, novelty preference; RT, response time; VPC, visual paired comparison.
