# Supplementary material for: Early Child Development Outcomes of a Randomized Trial Providing 1 Egg Per Day to Children Age 6 to 15 Months in Malawi
Source: J Nutr. 2020 Apr 14;150(7):1933–42. doi: 10.1093/jn/nxaa088 (PMC7330477; doi:10.1093/jn/nxaa088)
Supplement: nxaa088_Supplemental_File [file nxaa088_supplemental_file.docx]

“The effect of feeding children eggs on early child development in the Mazira Project randomized controlled trial in Malawi”

by

Elizabeth L. Prado, Kenneth Maleta, Bess L. Caswell, Matthews George, Lisa M. Oakes, Michaela C. Debolt, Megan G. Bragg, Charles D. Arnold, Lora Iannotti, Chessa Lutter, Christine P. Stewart

Online Supplementary Material

Supplemental Methods

Elicited Imitation Task

The elicited imitation task was adapted and piloted for use in the Mazira project in June to August, 2018. Three phases of piloting were completed, each with 10 children aged 12-15 months recruited from the community. After each phase, the task was assessed qualitatively and quantitatively. Qualitative assessment included feasibility of the test under the constraints of the research project, appropriateness of the test in the opinion of staff and caregivers, and acceptance of the objects by the infants. Quantitative assessment included basic analysis of the scores (mean, median, distribution, correlation with age) to determine if the test difficulty was appropriate. Adaptations made to the tasks included incorporation of local materials (i.e., switching a teddy bear for a local doll) and alteration of task difficulty (i.e., asking children to dip rather than stir a spoon in a cup). After each assessment and adaptation phase, the staff were retrained and new pilot children were recruited.

Before data collection, staff completed tests of reliability in which three videos were scored individually and compared to the trainer’s scores, with 90% agreement. After data collection, videos of the elicited imitation task were double-scored by UC Davis undergraduates for 75/576 (13%) randomly selected children. Inter-rater agreement was excellent for the spontaneous (ICC = 0.91), actions (ICC=0.92), and sequences (ICC=0.90) scores.

At endline, 5% of item scores were missing. For children with missing item scores, we multiplied the percent correct out of items available by the maximum possible score to calculate their total score.

*Child’s Mood, Activity, and Interaction with Assessor*

The child’s mood during the developmental assessment was rated as positive (smiling/laughing or occasional smiles) or not positive (crying/inconsolable, occasional crying, changeable/mood swings, or no visible emotions). The child’s interaction with the assessor was rated as positive (friendly) or not positive (avoidant and withdrawn, clings to family member, hesitant/when approached will accept reluctantly, difficult to engage in tasks, or inappropriate approaches to assessor). The child’s activity level was rated as positive (active and maintains interest) or not positive (unarousable, sleepy, can hardly be awakened, sleepy but easily awake, does not spontaneously engage in activity, and awake but loses interest).

*Developmental Assessment Quality Control*

The six data collectors who administered the MDAT independently scored six children from videos of the MDAT session. The five data collectors who administered the HOME inventory independently scored seven children from videos of the HOME interview and home visit. For each data collector, we calculated the percent of item scores that agreed with the mode value and averaged these across data collectors. For the elicited imitation task, 13% of videos were independently scored by a separate team of interns at UC Davis. For each data collector, we calculated the percent of item scores that agreed with the independent scorer, and averaged these across data collectors.

*Eye-Tracking Methods*

One laptop was a Dell Latitude 5480 and the other was a Dell Latitude 7280. Both monitors were HP Elite Display E222 21.5” monitors with 1920 X 1080 resolution.

The pilot version of the visual paired comparison task consisted of eight trials. The stimuli in the first four trials were based on the shapes used in Rose (1), for example, a star, a clover and a triangle. The shapes were grouped into pairs and one shape in each pair was designated as the familiar shape and the other as the novel shape. Each pair of shapes was a different color, and both shapes in the same pair were the same color. The shapes moved back and forth slowly on the screen, to simulate the procedure described in Rose (1), in which the experimenter moved the objects back and forth out of the infant’s reach. The stimuli in the next four trials were African faces from the database reported in Strohminger (2). Clips of classical music played while the shapes and faces were on the screen.

In each trial, a familiarization period was followed by a 20-second test for visual recognition memory. In each period, two stimuli were presented on the left and right sides of the screen. During the familiarization period, the same stimulus was presented on both sides. Following Rose (1), the familiarization period was 10, 15, 20, or 30 seconds. Each of these four familiarization times was used for one shape pair and one face pair. During the visual recognition memory period, the stimulus presented during the familiarization period appeared on one side and a novel stimulus appeared on the other side; the stimuli were reversed after the first 10 seconds.

The pilot data revealed that the looking time on the shape trials was low, demonstrating that the shapes did not capture the children’s attention. We therefore removed the shape trials and retained the face trials in the final version. Pilot data also revealed that few children looked at the faces for longer than 20 seconds of familiarization, therefore we used 20 seconds of familiarization time for all trials in the final version.

Statistical Analyses

For the eye-tracking scores, we conducted a secondary analysis of change from baseline to endline for the continuous primary outcomes only. These analyses only included the sub-set of children tested on the final version of the eye-tracking tasks at both baseline and endline. We tested the significance of the effect of the intervention on the change from baseline to endline by examining the time by group interaction. To each of the models for the primary analysis, we added a fixed effect of time and the time by group interaction. If the interaction was significant (p<0.1), we examined the effect of time stratified by intervention group and the effect of intervention group stratified by time.

Supplemental Results

A total of 215 children were tested on the final version of the eye-tracking task at both baseline and endline. For the three primary outcomes, the time by group interactions were not significant (*p*s>0.2), therefore we did not perform stratified analyses examining differences in change from baseline to endline between groups.

Supplemental References

1. Rose SA. Differential rates of visual information processing in full-term and preterm infants. Child Dev 1983;54(5):1189-98.

2. Strohminger N, Gray K, Chituc V, Heffner J, Schein C, Heagins TB. The MR2: A multi-racial, mega-resolution database of facial stimuli. Behav Res Methods 2016;48(3):1197-204. doi: 10.3758/s13428-015-0641-9.

3. Coates J, Swindale A, Bilinsky P. Household Food Insecurity Access Scale (HFIAS) for Measurement of Household Food Access: Indicator Guide (v. 3). Washington, D.C: Food and Nutrition Technical Assistance Project, Academy for Educational Development, 2007.

4. Caldwell BM, Bradley RH. Home Observation for Measurement of the Environment: Administration Manual. Tempe, AZ: Family & Human Dynamics Research Institute, Arizona State University, 2003.

*Supplemental Table 1*. Characteristics of participants included in analysis of MDAT and EI scores, compared to those not included.^1^

| Characteristics |  | | | Included in analysis | | | | | | Not included in analysis | | | | | |  |
| --- | --- | --- | --- | --- | --- | --- | --- | --- | --- | --- | --- | --- | --- | --- | --- | --- |
|  |  | | | N | | |  | | | N | | |  | | | p-value |
| Maternal |  | | |  | | |  | | |  | | |  | | |  |
| Maternal age, years | | | 585 | | | 26.0 ± 6.7 | | | 71 | | | 25.4 ± 7.5 | | | 0.42 | |
| Maternal BMI, kg/m^2^ | | | 585 | | | 21.8 ± 3.1 | | | 75 | | | 21.6 ± 2.6 | | | 0.56 | |
| Maternal education ≥ primary, % | | 585 | | | 20.9 | | | 77 | | | 13.0 | | | 0.09 | | |
| Mother can read, % | | | 582 | | | 47.1 | | | 61 | | | 34.4 | | | 0.06 | |
| Maternal marital status, % | | 585 | | |  | | | 77 | | |  | | | 0.09 | | |
|  | Monogamous | | |  | | | 56.8 | | |  | | | 66.2 | | |  |
|  | Polygamous | | |  | | | 19.8 | | |  | | | 20.8 | | |  |
|  | Unmarried | | |  | | | 23.4 | | |  | | | 13.0 | | |  |
| Child |  | | |  | | |  | | |  | | |  | | |  |
| Child age, months | | | 585 | | | 7.4 ± 1.2 | | | 77 | | | 7.5 ± 1.1 | | | 0.24 | |
| Female, % |  | | | 585 | | | 47.7 | | | 75 | | | 53.3 | | | 0.36 |
| Firstborn, % |  | | | 584 | | | 27.7 | | | 75 | | | 26.7 | | | 0.84 |
| Malaria, % |  | | | 529 | | | 12.7 | | | 68 | | | 11.8 | | | 0.83 |
| Anemia, % |  | | | 519 | | | 60.9 | | | 65 | | | 60.0 | | | 0.89 |
| Breastfeeding, % | | | 584 | | | 99.8 | | | 75 | | | 100.0 | | | 0.62 | |
|  |  | | |  | | |  | | |  | | |  | | |  |
| Household |  | | |  | | |  | | |  | | |  | | |  |
| Health center, % | | 585 | | |  | | | 77 | | |  | | | <0.01 | | |
|  | Lungwena | | |  | | | 50.6 | | |  | | | 74.0 | | |  |
|  | Malindi | | |  | | | 49.4 | | |  | | | 26.0 | | |  |
| Number of children under 5 years | | 577 | | | 1.7 ± 0.8 | | | 61 | | | 1.7 ± 0.8 | | | 0.95 | | |
| Moderate or severe food insecurity^2^, % | | | 585 | | | 77.3 | | | 75 | | | 82.7 | | | 0.28 | |
| Own latrine, % | | | 582 | | | 96.6 | | | 61 | | | 95.1 | | | 0.57 | |
| Distance to water source <10 min, % | | 582 | | | 55.5 | | | 61 | | | 57.4 | | | 0.78 | | |

^1^Values are N, %, or mean ± SD. BMI: body mass index; EI: Elicited Imitation; MDAT: Malawi Developmental Assessment Tool

^2^Food insecurity assessed using the Household Food Insecurity Access Scale (3)

*Supplemental Table 2*. Characteristics of participants included in analysis of eye-tracking scores, compared to those not included. ^1^

| Characteristics |  | | Included in analysis | | | | Not included in analysis | | | |  | |
| --- | --- | --- | --- | --- | --- | --- | --- | --- | --- | --- | --- | --- |
|  |  | | N | |  | | N | |  | | p-value | |
| Maternal |  | |  | |  | |  | |  | |  | |
| Maternal age, years |  | | 475 | | 26.0 ± 6.6 | | 181 | | 25.9 ± 7.2 | | 0.88 | |
| Maternal BMI, kg/m^2^ |  | | 475 | | 21.9 ± 3.1 | | 185 | | 21.7 ± 2.9 | | 0.53 | |
| Maternal education ≥ primary, % | | 475 | | 22.5 | | 187 | | 13.4 | | <0.01 | |  |
| Mother can read, % |  | | 473 | | 49.5 | | 170 | | 35.9 | | <0.01 | |
| Maternal marital status, % | | 475 | |  | | 187 | |  | | 0.51 | |  |
|  | Monogamous | |  | | 57.1 | |  | | 59.9 | |  | |
|  | Polygamous | |  | | 19.6 | |  | | 20.9 | |  | |
|  | Unmarried | |  | | 23.4 | |  | | 19.3 | |  | |
| Child |  | |  | |  | |  | |  | |  | |
| Child age, months |  | | 475 | | 7.4 ± 1.2 | | 187 | | 7.4 ± 1.1 | | 0.97 | |
| Female, % |  | | 475 | | 49.9 | | 185 | | 44.3 | | 0.20 | |
| Firstborn, % |  | | 474 | | 27.6 | | 185 | | 27.6 | | 0.99 | |
| Malaria, % |  | | 428 | | 14.0 | | 169 | | 8.9 | | 0.08 | |
| Anemia, % |  | | 420 | | 59.8 | | 164 | | 63.4 | | 0.42 | |
| Breastfeeding, % |  | | 474 | | 99.8 | | 185 | | 100.0 | | 0.42 | |
| Length-for-age z-score |  | | 475 | | -0.9 ± 1.0 | | 185 | | -1.0 ± 0.9 | | 0.16 | |
| Weight-for-length z-score |  | | 475 | | 0.1 ± 1.0 | | 185 | | 0.0 ± 1.1 | | 0.84 | |
| MDAT overall score |  | | 468 | | 43.5 ± 5.7 | | 185 | | 43.4 ± 5.7 | | 0.83 | |
|  |  | |  | |  | |  | |  | |  | |
| Household |  | |  | |  | |  | |  | |  | |
| Health center |  | | 475 | |  | | 187 | |  | | <0.01 | |
|  | Lungwena | |  | | 48.2 | |  | | 66.3 | |  | |
|  | Malindi | |  | | 51.8 | |  | | 33.7 | |  | |
| HOME inventory score^2^ |  | | 473 | | 24.2 ± 3.5 | | 170 | | 24.2 ± 3.6 | | 0.90 | |
| Number of children under 5 years | | 468 | | 1.7 ± 0.8 | | 170 | | 1.7 ± 0.8 | | 0.78 | |  |
| Moderate or severe food insecurity^3^, % | | 475 | | 76.6 | | 185 | | 81.1 | | 0.21 | |  |
| Own latrine, % |  | | 473 | | 97.3 | | 170 | | 94.1 | | 0.07 | |
| Distance to water source <10 min, % | | 473 | | 53.5 | | 170 | | 61.8 | | 0.06 | |  |

^1^Values are N, %, or mean ± SD. Malawi Developmental Assessment Tool

^2^Home Observation for Measurement of the Environment (4).

^3^Food insecurity assessed using the Household Food Insecurity Access Scale (3).

*Supplemental Table 3*. Mean differences in MDAT raw scores between 6-9 mo old children who did (Egg) or did not (Control) receive one egg per day for 6 mo^1^

|  |  | Baseline | | | | |  | Endline | | | | |  | Minimally adjusted^2^  comparison (95% CI) | Fully adjusted^3^  comparison (95% CI) |
| --- | --- | --- | --- | --- | --- | --- | --- | --- | --- | --- | --- | --- | --- | --- | --- |
|  |  | Control | |  | Egg | |  | Control | |  | Egg | |  |  |  |
|  |  | N |  |  | N |  |  | N |  |  | N |  |  |  |  |
|  | Fine motor score | 323 | 12.45 ± 2.47 |  | 329 | 12.31 ± 2.40 |  | 293 | 16.49 ± 2.49 |  | 284 | 16.66 ± 2.62 |  | 0.25  (-0.16, 0.67) | 0.09  (-0.26, 0.44) |
|  | Gross motor score | 323 | 12.86 ± 1.76 |  | 329 | 12.66 ± 1.84 |  | 293 | 17.79 ± 3.16 |  | 284 | 17.54 ± 2.79 |  | -0.16  (-0.60, 0.27) | -0.30  (-0.72, 0.11) |
|  | Language score | 323 | 6.57 ± 1.48 |  | 329 | 6.56 ± 1.43 |  | 293 | 10.41 ± 1.73 |  | 284 | 10.53 ± 1.86 |  | 0.14  (-0.14, 0.41) | -0.08  (-0.35, 0.18) |
|  | Personal-social score | 323 | 11.73 ± 2.19 |  | 329 | 11.80 ± 1.97 |  | 293 | 16.68 ± 3.33 |  | 284 | 16.82 ± 3.20 |  | 0.14  (-0.36, 0.64) | 0.10  (-0.31, 0.52) |

^1^Values are N, mean ± SD, or mean difference (95% CI); MDAT: Malawi developmental assessment tool

^2^Adjusted for baseline MDAT scores.

^3^Adjusted for variables in the minimally adjusted model and potentially adjusted for child age at measurement, sex, birth order, maternal age, height, education, literacy, marital status, tribe, occupation, religion, number of children under 5 y in the household, food security, housing and asset index, animal ownership, distance to water source, closest health center, length-for-age z-score, weight-for-length z-score, HOME inventory score, field staff who performed measurements, month of measurement, time of day of measurement, child's demeanor during measurement, and endline family care indicator score. Additionally the language outcomes are potentially adjusted for child's primary language and exposure to multiple languages.

*Supplemental Table 4*. Results of analysis of potential modifiers of the effect of the intervention.

|  | Effect Modifier | | | | | | | | | |
| --- | --- | --- | --- | --- | --- | --- | --- | --- | --- | --- |
| Outcome | Sex | Firstborn child | Maternal age | Maternal education | Food insecurity | Household asset index | Baseline LAZ < -1 | Corresponding baseline developmental score | HOME Inventory score | FCI score |
| Fine motor norm z-score | ns^1^ | Positive effect in later-born | Positive effect in children of mothers > 20 y | ns | ns | ns | Positive effect in children with LAZ ≥ -1 | ns | ns | ns |
| Gross motor norm z-score | ns | ns | ns | ns | ns | ns | ns | ns | ns | ns |
| Language norm z-score | ns | ns | ns | ns | Positive effect in households with mild to no food insecurity | ns | ns | ns | ns | ns |
| Personal-social norm z-score | ns | ns | ns | ns | ns | Positive effect in households in the highest wealth quintile | ns | ns | ns | ns |
| Elicited imitation total actions recalled | ns | ns | ns | Positive effect in children of mothers with primary or greater education | ns | ns | ns | ns | ns | ns |
| VPC novelty preference | Positive effect in girls | ns | ns | ns | ns | ns | ns | ns | ns | ns |
| VPC mean familiarization fixation | ns | ns | ns | ns | ns | ns | ns | ns | ns | ns |
| IOWA response time | ns | ns | ns | ns | ns | ns | ns | ns | ns | ns |

^1^ns: non-significant p-value for the interaction term between the effect modifier and intervention group (p > 0.05)

*Supplemental Figure 1*. Data collection at the field site using an automated eye tracker

*
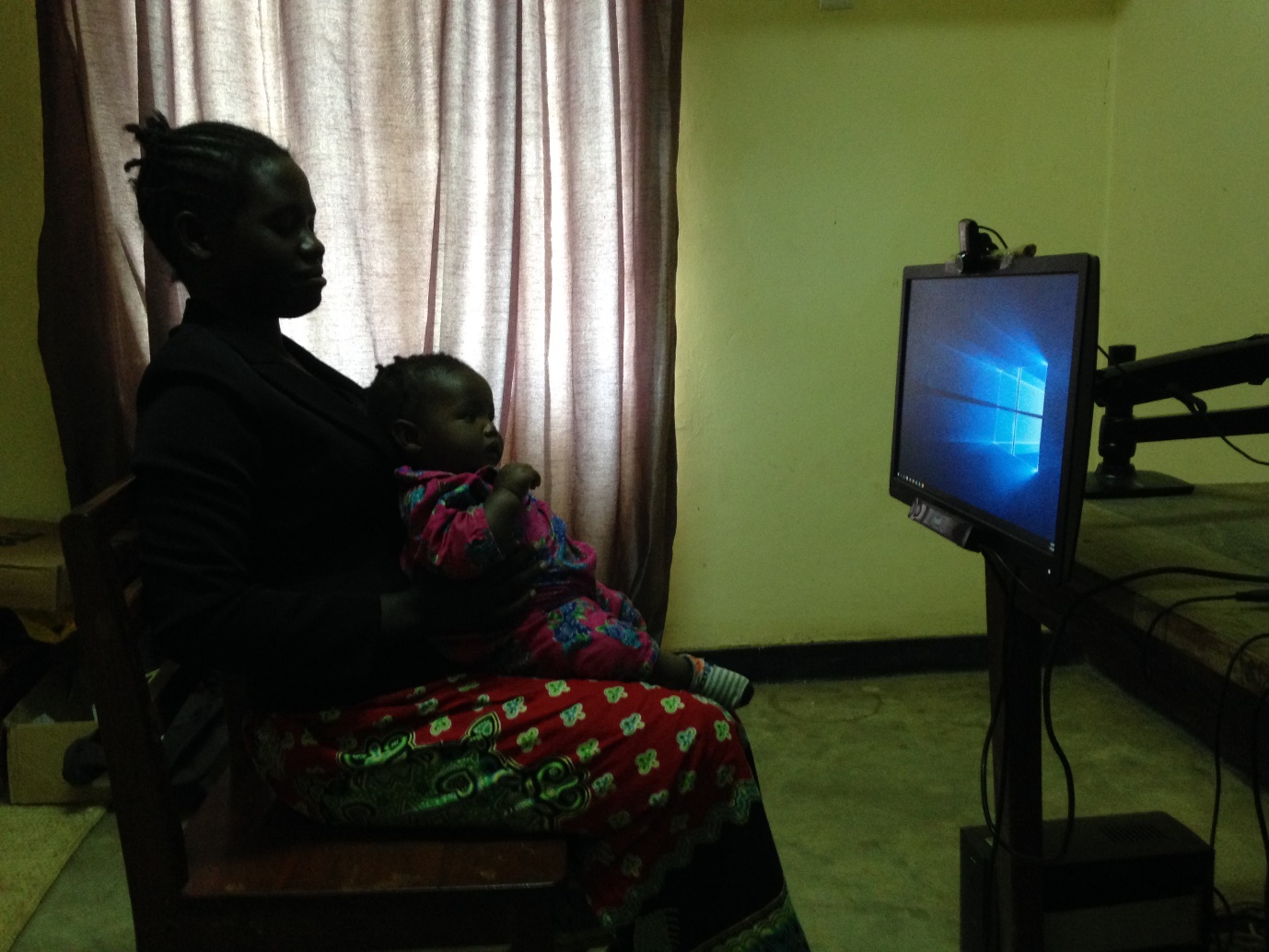
*

*Supplemental Figure 2.* Standardized mean differences between 6-9 mo old children who did (Intervention) or did not (Control) receive one egg per day for 6 mo stratified by each significant effect modifier.

*
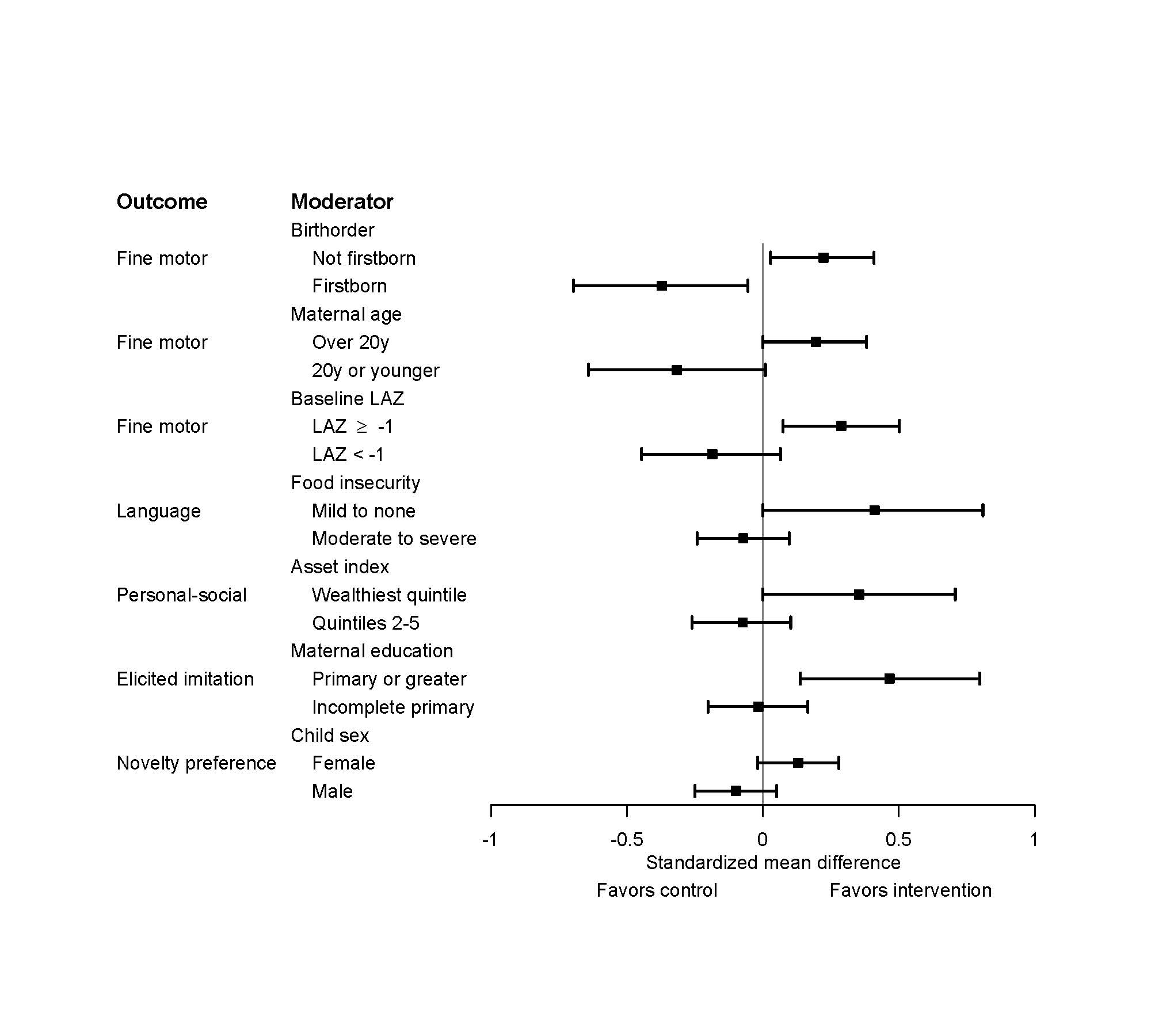
*
